# Supplementary material for: Development and evaluation of a modular smoke evacuator for surgical smoke control
Source: PLoS One. 2026 Jan 16;21(1):e0341110. doi: 10.1371/journal.pone.0341110 (PMC12810818; doi:10.1371/journal.pone.0341110)
Supplement: Attachment — Submitted filename: Response to Editor and Reviewers.docx [file pone.0341110.s001.docx]

**Response to Editor and Reviewers**

We would like to sincerely thank the Academic Editor and all reviewers for their insightful, constructive, and encouraging comments. Their feedback has been invaluable in improving the clarity, methodological transparency, and scientific strength of our manuscript.

All revisions have been incorporated accordingly, and the corresponding changes are highlighted in yellow throughout the revised manuscript.

**Response to Academic Editor**

We are deeply grateful to the Editor for recognising the innovation and potential merit of our experimental model. In response to the Editorial recommendation, we have substantially enhanced the *Engineering Description* as pointed out by Reviewer 2, with particular focus on the *Integration with the Laparoscopic System*.

A detailed description of the airflow pathway, three-way valve configuration, and passive evacuation mechanism has been added in the *Methods* section. In addition, a schematic figure and an explanatory paragraph in the *Discussion* were included to emphasise system reliability and pressure stability during laparoscopic use.

The Editor also highlighted the importance of addressing the *generalisability of the experimental model to clinical practice*. This has been revised and clearly discussed under the *Limitations* section, explaining that the closed-chamber setup represents a validated pre-clinical model consistent with previous literature (e.g., Sadowsky et al., 2023) and serves as a necessary step before live surgical validation.

Finally, the new reference suggested by Reviewer 3 has been carefully reviewed and incorporated in the *Discussion* as comparative evidence. The manuscript now includes explicit statements bridging the bench-top results to future clinical translation.

**Response to Reviewer #1**

We sincerely thank Reviewer #1 for the encouraging and positive evaluation.

We are truly grateful that you found the study design interesting and publication-worthy. Your remarks reinforce the potential value of low-cost, modular smoke evacuation systems, especially for resource-limited settings. No specific revisions were required, but we have ensured that all technical and stylistic aspects have been refined to maintain scientific clarity and conciseness.

Once again, we greatly appreciate your supportive review and kind recommendation for publication.

**Response to Reviewer #2**

We deeply appreciate Reviewer #2’s comprehensive and highly constructive technical feedback. Your insightful comments have significantly strengthened the engineering and methodological aspects of our work. Revisions made in response to your points are highlighted in yellow throughout the revised manuscript.

**1. Incomplete Engineering Description**

We have expanded the *Prototype Device Development* section to include complete details on the suction mechanism, airflow dynamics, and system configuration.

The revised text specifies the tubing dimensions, pressure resistance, estimated flow rate (8–10 L/min), and airflow direction across the three-stage filtration circuit.

Additionally, a new paragraph in the *Discussion* elaborates on the passive flow design and confirms that flow testing demonstrated stable evacuation without turbulence or reverse flow.

**2. Unclear Integration with the Laparoscopic System**

This issue has been thoroughly addressed.

A new subsection entitled *Integration with Laparoscopic System* now explains the connection between the evacuator and laparoscopic ports through a three-way valve, maintaining pneumoperitoneum integrity.

A schematic figure was added for clarity. The *Discussion* also emphasises that the system operates without a continuous suction motor, preserving intra-abdominal pressure and workflow continuity during surgery.

**3. Simplified Experimental Model and Generalisability**

We fully acknowledge this important concern.

The *Limitations* section now explicitly states that the sealed acrylic chamber represents a simplified pre-clinical model aligned with prior studies (e.g., Sadowsky et al., 2023).

We clarified that this controlled environment allows quantitative reproducibility before clinical trials. A statement has been added noting that future studies will include live surgical validation under realistic OR airflow conditions.

We are sincerely thankful for these comments, which helped us refine our technical presentation and clarify the translational potential of the work.

**Response to Reviewer #3**

We are very grateful for Reviewer #3’s generous comments and thoughtful suggestions, which helped strengthen both the discussion and contextual grounding of our study.

All relevant revisions are highlighted in yellow in the revised manuscript.

**1. Additional Reference**

We have reviewed the cited work by *Mordechai G. Sadowsky et al., 2023* and found it highly relevant. It has been incorporated into the *Discussion* in three locations:

(1) as supportive evidence for particulate and formaldehyde removal performance,

(2) to compare noise and practical usability aspects, and

(3) in the *Limitations* section to justify the use of controlled chamber simulations in early-phase studies.

**2. Clarification Regarding CO₂ Levels**

We appreciate your keen observation.

A sentence has been added to the *Discussion* explaining that the observed CO₂ stability was due to the absence of CO₂ capture by filters and the dilution effect from chamber ventilation. We also clarified that the system was not intended to absorb CO₂, consistent with its role in laparoscopic pneumoperitoneum maintenance.

We are sincerely thankful for your insightful analysis and valuable recommendations that enriched the scientific discussion of our study.

Once again, we extend our deepest gratitude to the Editor and all three reviewers for their exceptional feedback.

Your critical and encouraging comments have helped us substantially improve the manuscript’s clarity, scientific rigor, and translational focus.

We believe the revised version now addresses all concerns comprehensively and is substantially strengthened for publication consideration.

All revisions have been highlighted in yellow in the revised manuscript for your convenience.

With our highest respect and appreciation,

On behalf of all authors,

*Suphakarn Techapongsatorn, MD, PhD.*

Faculty of Medicine Vajira Hospital

Navamindradhiraj University
